# The Yorkshire College, Leeds

**Published:** 1889-10-12

**Authors:** 


THE YORKSHIRE COLLEGE, LEEDS.

Dr. W. T. Gairdner gave an address which a contemporary designates "A plea for thoroughness." He commenced with an account of an automatic machine latterly invented, in the shape of a wooden figure like a man. The figure is covered with compartments labelled with the names of various ailments. The person requiring aid places a piece of money in the compartment with the name of his illness (a difficult matter to attain sometimes), and forthwith appears a pill or powder suited to the case! In this physic-loving England, we should imagine, there will be a run on these machines, if placed in public places, as we have the ordinary automatic chocolate, bonbon, scent, and match machines.

The lecturer, however, does not imagine that such a machine could even be made a clever doctor! For to treat disease "real knowledge" is required, and not a "rule o' thumb" treatment; this for that and that for this. "When I hear," says Dr. Gairdner, "of a man talking large and dogmatically about biliousness (a term of Abernethy's which neither Abernethy nor anyone else has been able to explain further than that it requires blue pill), or about congestion of the brain or anæmia of the same, or about irritation of the mucus membrane, he is using words, not to set forth knowledge, but to conceal ignorance." Still these names, and many others which Dr. Gairdner would include in the same category, such as "hyperœsthia," "hyperœmia," "neurasthenia," are, and will be, used by physicians. For although, as Dr. Gairdner says, they may not be known as facts, still they imply a certain meaning and condition, and we should be glad to learn if Dr. Gairdner himself does without their use. It is all very well to "try to think of diseased phenomena as men do who have and who use the means of knowing them as facts," but if we are to allow nothing in medicine but what we know as facts, we should cease to allow many things which have been theoretically reasoned out, until they seem almost as certain as the exact sciences. For instance, we cannot see either congestion or anæmia of the brain during life, and therefore we cannot know that it really exists in life; but we have a certain set of symptoms characteristic of either condition, and we find such condition after death, so that we cannot agree with Dr. Gairdner that there are terms without a meaning. The gist of the lecturer's address was that every student should obtain a knowledge of things as opposed to words and abstract ideas. But we must have words and abstract ideas, for without them we cannot express our knowledge of things fully, neither can we apply that knowledge to practical purposes. Like the St. George's lecturer, the Leeds lecturer does not agree with Mr. Wheelhouse's views of more practical teaching for the student before he commences to "walk the hospitals." Dr. Gairdner takes care that *his* students shall not be ignorant of measles and common disorders. But we fully believe there is no school so good for a young man as a few months in the surgery of a general practitioner, for he learns more of the routine of practice in such a position in a few months than during years in a hospital; and private practice and hospital practice are somewhat different, as not a few have found when having "walked the hospitals," and got a diploma, they essay to doctor the public on their own account.

